# Using intervention mapping to evaluate ‘High-Alert,’ a brief smartphone intervention to reduce youth cannabis-impaired driving

**DOI:** 10.1371/journal.pone.0329383

**Published:** 2025-08-22

**Authors:** Robert Colonna, Patricia Tucker, Angela Mandich, Liliana Alvarez

**Affiliations:** 1 Health and Rehabilitation Sciences, Western University, London, Ontario, Canada; 2 School of Occupational Therapy, Western University, London, Ontario, Canada; 3 Lawson Health Research Institute, London, Ontario, Canada; 4 Children’s Health Research Institute, London, Ontario, Canada; NYU Grossman School of Medicine: New York University School of Medicine, UNITED STATES OF AMERICA

## Abstract

Youth driving under the influence of cannabis (DUIC) is a growing public health concern. While brief smartphone interventions have shown promise in reducing substance use and alcohol-impaired driving among youth, their efficacy for DUIC remains limited. Using the six-step Intervention Mapping framework, we developed and tested High Alert, a digital smartphone intervention designed to reduce DUIC among high-risk Canadian youth. The intervention was previously tested in a pilot randomized controlled trial comparing High Alert to an active control (exposure to six DUIC infographics) and a passive control (no contact). This study presents a comprehensive evaluation of High Alert using Step 6 of the Intervention Mapping framework. Reporting on this evaluation serves as a practical guide for researchers utilizing Intervention Mapping, offering valuable insights into High Alert’s formative, process, outcome, and acceptability evaluations to enhance DUIC prevention efforts. Formative and acceptability evaluations revealed High Alert’s positive reception among youth, with most participants willing to engage with it and recommend it to their peers. The program received high ratings for content and delivery, surpassing the static infographics used in the active control. Outcome evaluations demonstrated preliminary efficacy in reducing DUIC behaviour, particularly driving after cannabis co-use, compared to the no-contact group. Process evaluations highlighted implementation challenges, including online study bot activity, recruitment barriers (e.g., participant skepticism, limited ad targeting options), high attrition rates, and low adherence. Findings highlight the importance of Step 6 in Intervention Mapping, emphasizing the need for transparent and rigorous evaluation to inform future interventions. Addressing recruitment and implementation challenges is essential for improving the scalability and effectiveness of interventions targeting high-risk behaviours such as DUIC and will inform High Alert’s future testing.

## Background

With the legalization of recreational cannabis in Canada and across several U.S. states, there are growing concerns regarding driving under the influence of cannabis (DUIC), particularly among youth [1,2]. Recent evidence suggests that young drivers (ages 16–24 years) engage in the highest rates of cannabis use and DUIC [3,4], and express the least concern towards this risky behaviour [5]. National fatality data reveals that 38% of drivers aged 16–19 and 41% of those aged 20–24 killed in crashes tested positive for cannabis [6]. These rates surpass the 28.3% of all drivers testing positive for cannabis post-legalization (2018–2021) and the 23.3% pre-legalization (2015–2018), suggesting an upward trend [7]. Given that cannabis impairs youth driving performance [8] and increases crash risk [9,10], increasing attention is being paid to strategies aimed at reducing DUIC among young drivers. This includes the growing use of brief digital and smartphone-based approaches [11–16], especially as many DUIC educational programs have shifted online due to the COVID-19 pandemic [17].

For decades, brief educational interventions delivered face-to-face or via computers have shown efficacy in reducing youth substance use and alcohol-impaired driving [18–22]. These brief interventions draw on behaviour change principles to increase perception of risk, enhance self-efficacy, correct misperceptions of descriptive norms, provide personalized feedback, offer normative comparisons, challenge positive alcohol expectancies, and deliver tailored education [19,22,23]. Smartphone-delivered brief interventions applying these principles are gaining popularity for their wider reach, easier access, and enhanced availability and replicability [23,24]. Preliminary evidence from the United States suggests that brief smartphone interventions can reduce alcohol-impaired driving and DUIC among youth [11,20,25,26]. However, additional evidence is needed to develop and test these initiatives for young Canadians [17].

In response to this need, the *High Alert* intervention was developed using the 6-step Intervention Mapping framework [27] to reduce DUIC among Canadian youth. The Intervention Mapping framework is common in the substance use literature [28,29] and includes the following steps: (1) conducting a needs assessment, (2) identifying intervention objectives, (3) selecting intervention theory-based methods and practical strategies, (4) developing intervention components and materials, (5) planning and executing the initial implementation, and (6) evaluating the intervention [27]. A detailed description of High Alert’s development (Steps 1–4) is reported elsewhere [30], with findings of the initial implementation (Step 5) providing initial support for High Alert’s potential to reduce DUIC behaviours compared to a no-contact control group [31]. The current paper describes Step 6, detailing the findings from the intervention’s systematic evaluation.

Extensive literature underscores the value of Intervention Mapping Steps 1–5, providing insights into program development, theoretical foundations, implementation, and primary outcomes of health promotion programs [28–30,32–43]. However, fewer studies rigorously document Step 6, a necessary evaluation process that reflects the intervention’s fidelity, outcomes, and acceptability, among other components [28–30,32–43]. In fact, the product of Step 6 is a comprehensive evaluation that informs program improvements before implementation (formative evaluation), monitors the intervention’s implementation and fidelity (process evaluation), determines if the final program met its goals and objectives (outcome evaluation), and assesses program acceptability, facilitating future implementations [27,44].

Reporting on this final step is particularly important for interventions addressing high-risk behaviours such as cannabis use and DUIC, like High Alert. While challenges such as participant engagement, attrition, and fidelity are common to many health promotion interventions, digital interventions often face additional complexities, including technical issues, digital literacy barriers, and risks of contamination in virtual settings [24,45,46]. Moreover, digital interventions must comply with several regulatory, ethical, and data protection standards [24], especially when participants disclose criminal behaviour such as DUIC (e.g., as in [31]). Evidence demonstrates that interventions with fidelity assessments are more effective in reducing youth cannabis use [47], yet few smartphone-based programs targeting substance use and impaired driving have been rigorously evaluated, with some exacerbating harmful use [15,16,48]. In this context, evaluation is essential for the High Alert program to support ongoing program improvement, assess initial implementation and fidelity, identify mechanisms of change, and ensure participant acceptability, all of which can inform future testing and implementations.

Thus, this study aimed to conduct a comprehensive evaluation of High Alert using Step 6 of the Intervention Mapping framework. Reporting on this evaluation acts as a practical guide for researchers utilizing Intervention Mapping and offers valuable insights into High Alert’s formative, process, outcome, and acceptability evaluations to enhance DUIC prevention efforts.

## Methods

### Study design

This study followed Step 6 of the Intervention Mapping framework to conduct a comprehensive evaluation of High Alert’s implementation. The High Alert program was evaluated in a three-arm pilot-randomized controlled trial (ClinicalTrials.gov; NCT06098573) comparing High Alert to an active control (six infographics on cannabis and DUIC) and a passive control (no contact) [31]. Participants were recruited via social media and mass-email recruitment from November 2023 to June 2024 and included Ontario drivers aged 18–24 who owned a smartphone, held a valid driver’s license, had access to a vehicle, and reported DUIC three or more times in the past three months [11,49]. Ethics approval was obtained from Western University’s Research Ethics Board (ID #123368), and all participants provided written informed consent prior to participating.

### High Alert program

High Alert consists of four interactive e-learning modules that educate youth on: (1) the effects of cannabis on driving, (2) the risks of DUIC, (3) strategies to avoid DUIC, and (4) risky cannabis use. Module content was tailored to young Ontario drivers and informed by previous studies exploring youth substance use, DUIC, and related interventions [12,13,50–52]. The program is delivered over two weekly sessions (10 minutes each) using the Computerized Intervention Authoring System (CIAS, [53]). A comprehensive overview of each module’s content, theoretical foundations, and design is reported in [30], and full details on the pilot trial (e.g., recruitment, randomization, trial arms) and the primary and secondary outcome measures are reported in [31].

### Data collection

Data were collected online through Qualtrics Survey Software and the CIAS platform (i.e., usage data) [53]. Measurements were assessed during the pre-testing phase and in the pilot trial at baseline, post-intervention, and 3-month follow-up. A 3-month timeframe was chosen to align with previous research and enable outcome comparison [11,20,25,26,49]. Table 1 provides an overview of measurement administration.

**Table 1:** Study measurement administration schedule.

Construct	Pre-baseline	Baseline	Post-intervention	3-month follow-up
Pre-testing (before pilot trial)	X			
High Alert usage data		X		
Screening & Consent		X		
Demographics		X		
Cannabis Use (CUDIT-R)		X		X
Cannabis & Driving History		X		
Cannabis & DUIC		X		X
Knowledge		X	X	
DUIC Perceptions		X	X	X
Contextual factors		X		X
DUIC Normative Beliefs		X	X	X
Program Feedback & Acceptability	X		X	

DUIC = driving under the influence of cannabis; CUDIT-R = Cannabis Use Disorder Identification Test-Revised.

### Evaluation components

**Fig 1:**
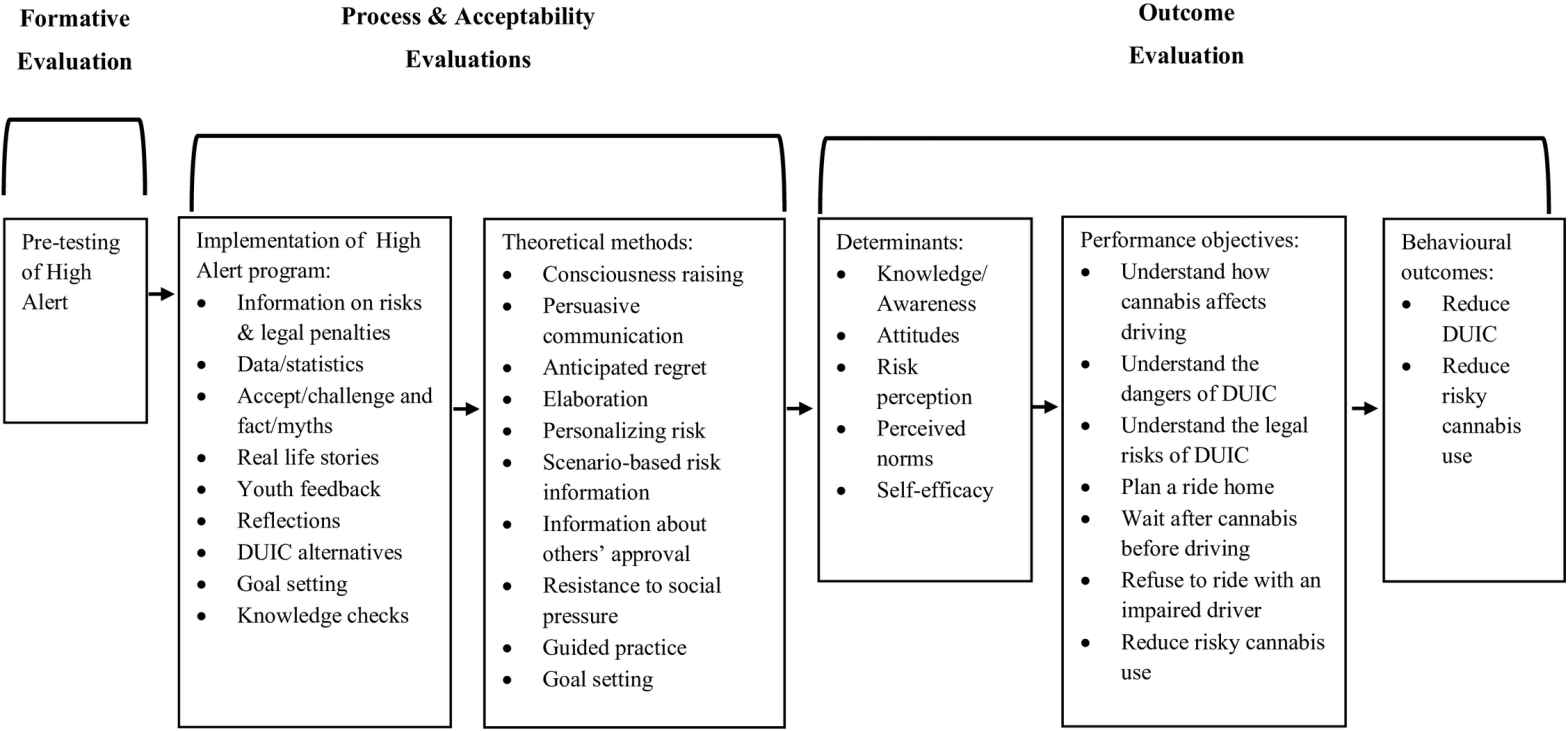
High alert logic model. DUIC = driving under the influence of cannabis.

The comprehensive evaluation covered formative, process, outcome, and acceptability components. Fig 1 displays the program logic model, providing a visual representation of the High Alert program and where each evaluation component fits within the overall framework. While the formative evaluation and the specific primary and secondary behavioural outcomes were previously reported [30,31], summaries are included here to provide a comprehensive overview of the evaluation plan and integration of findings. All other evaluation components are novel to this paper. Table 2 outlines the comprehensive evaluation plan.

**Table 2:** Evaluation plan: High alert program.

Question, Indicator	Measures	Source	Time point	Analysis [citation if previously reported]
Formative Evaluation				
*Pretesting – Each Module*				
Concepts	Was there any part of the *module* that you especially liked? Was there anything about the *module* that you disliked, found confusing, or hard to understand? (text responses)	Pretest survey	Pre-test	Text-based review ^a^
Content	“In your opinion, was the content in this *module*:” believable, convincing, interesting, informative, easy to understand, and personally relevant. (4 pt. scale from ‘Not at all’ to ‘Very’).	Pretest survey	Pre-test	Descriptive statistics ^a^
Feedback	“Please add any additional comments/feedback about this *module*” (text responses)	Pretest survey	Pre-test	Text-based review ^a^
*Pretesting – Entire Program*				
Feedback	“If you have any additional comments or feedback about the program, please write it below” (text responses)	Pretest survey	Pre-test	Text-based review ^a^
Readability	Flesch Reading Ease Score and Kincaid Grade Level	Program script	Pre-T1	Readability scores ^a^
Process Evaluation				
*Context*				
Knowledge seeking	“In the past 3 months, have you sought out any other information related to cannabis use or driving under the influence of cannabis” (Yes/No and text responses)	Online survey	T1, T3	Descriptive statistics ^b^ & Text-based review
Contextual factors	“In the past 3 months, has any personal, social, or environmental factors influenced how often you drive after cannabis?” (Yes/No and text responses)	Online survey	T1, T3	Descriptive statistics ^b^ & Text-based review
*Reach*	Participation rate, demographics of participants, pattens and causes for incomplete participants (e.g., differences in demographics and outcome variables)	Online survey & CIAS usage	T1, T2, T3	Descriptive statistics ^b^
*Dose Delivered*	The total number of modules started	CIAS usage	T2	Descriptive statistics
*Dose Received*	Total number of modules completed. Average time per session,	CIAS usage	T2	Descriptive statistics
*Fidelity*	Degree to which the intervention was delivered as intended (e.g., module order, session spacing) and engaged with (e.g., number of interactions, number of questions skipped, access issues).	CIAS usage & Trial protocol	T2	Descriptive statistics & CIAS usage data
*Recruitment*	Number of likes/shares on social media recruitment posts. Number of participants recruited form each source	Social media & screening	Pre-T1	Descriptive statistics
Outcome Evaluation				
*Behavioural Outcomes*				
DUIC behaviour	Number of times reporting DUIC (from cannabis alone) in the past 3 months (continuous)	Online survey	T1, T3	GLMM ^b^
Total DUIC behaviour	Number of times reporting Total DUIC (from cannabis alone plus instances when combined with other substances) in the past 3 months (continuous)	Online survey	T1, T3	GLMM ^b^
Risky cannabis use	CUDIT-R score using the past 3-month timeframe (continuous)	Online survey	T1, T3	ANOVA ^b^
*Behavioural Intentions*				
Expected DUIC after program completion	“After this program, how often do you think you will drive under the influence of cannabis?” (3 pt. scale from ‘Less than before’ to ‘More than before).	Online survey	T2	Mann-Whitney U test
Change in DUIC intent	“In the next 3 months, do you think that at least once you will drive within 2 hours of using cannabis?” (5-point Likert scale from ‘Definitely Not’ to ‘Definitely Yes’).	Online survey	T1, T3	Kruskal-Wallis H test
*Behavioural Determinants*				
Change in knowledge	Total number of correct responses on a 27-item knowledge scale (continuous)	Online survey	T1, T2	Mann-Whitney U test
Change in attitudes (moral wrongness)	“In your opinion, is driving under the influence of cannabis wrong?” (4 pt. scale from ‘Not at all” to ‘Always’).	Online survey	T1, T3	Kruskal-Wallis H test
Change in attitudes (acceptability)	“Do you think there are situations where driving under the influence of cannabis is acceptable?” (Yes/No)	Online survey	T1, T3	Kruskal-Wallis H test
Change in risk perception (danger)	“In your opinion, how dangerous is driving under the influence of cannabis?” (4 pt. scale from ‘Not at all” to ‘Very’).	Online survey	T1, T3	Kruskal-Wallis H test
Change in risk perception (legal)	“In your opinion, how likely would an individual driving under the influence of cannabis be involved in legal consequences” (3 pt. scale from ‘Not likely” to ‘Very likely’).	Online survey	T1, T3	Kruskal-Wallis H test
Change in perceived Norms	“My peers would appreciate it if I planned a ride home (e.g., taxi, rideshare, public transport) instead of driving high” pressure (3 pt. scale from ‘Agree” to ‘Disagree’).	Online survey	T1, T3	Kruskal-Wallis H test
Change in self-efficacy (avoiding DUIC)	“How confident are you that you can avoid driving after using cannabis in the next 3 months?” (4 pt. scale from ‘Not at all” to ‘Very’).	Online survey	T1, T3	Kruskal-Wallis H test
Change in self-efficacy (resisting peer pressure)	Sum of agreement to 3 items on resistance to peer pressure (3 pt. scale from ‘Agree” to ‘Disagree’. Scores range from 0 to 3, with higher scores indicating better resistance.).	Online survey	T1, T3	Kruskal-Wallis H test
Acceptability				
Program concepts	Was there any part of the *program* that you especially liked? Was there anything about the module that you disliked, found confusing, or hard to understand? (Yes/No and text responses)	Online survey	T2	Descriptive statistics & Text-based review
Program contents	“In your opinion, was the content in this *program*:” believable, convincing, interesting, informative, easy to understand, and personally relevant. (4-point Likert scale from ‘Not at all’ to ‘Very’).	Online survey	T2	Descriptive statistics & Mann-Whitney U tests
Program delivery	“In your opinion, was the software/ application used to deliver the program:” easy to use, engaging (4-point Likert scale from ‘Not at all’ to ‘Very’).	Online survey	T2	Descriptive statistics & Mann-Whitney U tests
Program recommendation	“Overall, how likely are you to recommend this program to your peers?” (5-point Likert scale from ‘Very Unlikely’ to ‘Very Likely’).	Online survey	T2	Descriptive statistics & Mann-Whitney U tests

T1 = Baseline; T2 = Post-intervention; T3 = 3-months post-intervention; DUIC = driving under the influence of cannabis; CIAS = Computerized Intervention Authoring System; GLMM = Generalized Linear Mixed Modelling; ANOVA = Analysis of Variance; CUDIT-R = Cannabis Use Disorder Identification Test Revised.

^a^previously reported in [30].

^b^previously reported in [31].

#### Formative evaluation.

The formative evaluation assessed the intervention’s components prior to implementation [27]. A pretest was conducted with five youths in the target age range and five adult cannabis educators (i.e., professionals with experience delivering Canadian cannabis educational programming) [30] to assess each module’s concepts, content (i.e., believable, convincing, interesting, informative, easy to understand, and personally relevant), and general program feedback [27,54]. Additionally, readability scores were calculated for the overall program. Methods and findings of this formative evaluation are presented in [30].

#### Process evaluation.

The process evaluation assessed High Alert’s implementation during the pilot trial, following the framework by Linnan and Steckler [55]. Key areas included context (the social and environmental conditions impacting implementation), reach (the percentage of the target audience that received High Alert), dose delivery (the amount of the program delivered), dose received (the amount of the program received and engaged with), fidelity (how closely High Alert was delivered as intended), and recruitment (how participants were recruited). This framework shares similarities with the RE-AIM framework (Reach, Effectiveness, Adoption, Implementation, Maintenance) [56] in its focus on key components such as reach, fidelity, and dose. However, it places more emphasis on process-oriented aspects like context, recruitment, dose delivered, and dose received, while the RE-AIM framework emphasizes broader components like effectiveness, adoption, and maintenance [57]. Data on context and some reach aspects were collected via baseline and 3-month follow-up questionnaires. CIAS usage data provided insights into other reach aspects, dose delivered, and dose received. Fidelity was evaluated with CIAS usage data, while recruitment data came from social media analytics (i.e., number of likes, shares, comments, post removals) and the screening survey.

#### Outcome evaluation.

The outcome evaluation assessed differences in behavioural outcomes, intentions, and effects on determinants following participation in the High Alert program [27]. Measures were adapted from the validated Youth Cannabis and Diving Survey [50] or created for this study.

##### Behavioural outcomes

The primary outcome was self-reported DUIC behaviour over the past 3 months, assessed at baseline and the 3-month follow-up. Two variables were analyzed (i) DUIC involving cannabis only, and (ii) total DUIC incidents, including cannabis co-use with alcohol/other drugs. Each was analyzed separately to avoid inflating DUIC counts from question misinterpretation (i.e., reporting the same instance across multiple categories; see [31]). The secondary outcome was risky cannabis use measured using the Cannabis Use Disorder Identification Test-Revised (CUDIT-R [58]). The scale was adapted to assess behaviour over the past 3 months (instead of 6), detecting hazardous use (score 8–11) and potential cannabis use disorder (score 12 + , maximum 32) [58].

##### Behavioural intentions

DUIC intentions were assessed using two questions: a post-intervention measure evaluating participants’ expected change in DUIC after program completion (rated on a 3-point scale from ‘less than before’ to ‘more than before’), and a change score that measures differences in future DUIC intent between baseline and 3-month follow-up (rated on a 5-point Likert scale from ‘definitely not’ to ‘definitely yes’) [50].

##### Behavioural determinants

Determinants of DUIC identified in Step 2 of Intervention Mapping included knowledge, attitudes, risk perception, perceived norms, and self-efficacy [30]. These determinants were identified from prior literature that explored DUIC behaviour using various theoretical frameworks, such as social learning theory [59], the theoretical framework of general deterrence and general prevention [50], the theory of planned behaviour [60], and the prototype willingness model [61]. Knowledge was assessed immediately post-intervention with a 27-item knowledge score. Attitudes refer to a person’s feelings toward an object, behaviour, or concept [62] and were measured with two questions gauging participants’ moral stance on whether DUIC is wrong and their beliefs about its acceptability [50]. Risk Perceptions relate to an individual’s subjective judgement on the perceived risks associated with a behaviour [63] and were evaluated with two questions that assess DUIC’s dangerousness [50,59,64,65] and likelihood of experiencing legal repercussions [50]. Perceived norms focused on the perceived value from peers on planning a safe ride home instead of DUIC, while self-efficacy assessed confidence in avoiding DUIC in the coming months and resistance to peer influence [50].

#### Acceptability evaluation.

The last evaluation assessed participants’ acceptability and reactions to High Alert post-intervention [27,54]. Participants rated the overall program content (i.e., believable, convincing, interesting, informative, easy to understand, and personally relevant) and delivery (i.e., ease of use, engagement) using a 4-point Likert scale from not at all to very. General feedback was solicited with open-ended questions asking participants about the program concepts (i.e., what they liked and disliked) and a question about program recommendations to peers.

### Data analysis

Table 2 outlines the data analysis methods for each evaluation variable. Analyses were conducted using IBM SPSS version 26 and R version 4.4.1, incorporating both descriptive (e.g., frequencies, means) and inferential statistics. Generalized Linear Mixed Modelling was used to assess group differences over time for the primary outcome variable (i.e., past DUIC), and a two-way mixed Analysis of Variance (ANOVA) was used to assess the secondary outcome (i.e., risky cannabis use). The Kruskal-Wallis H test was used for assessing change scores across three groups with ordinal (i.e., Likert scale) or non-normal data, while the Mann-Whitney U test was applied for comparisons between two groups under similar conditions. Participants’ written responses to optional open-ended questions were reviewed and presented descriptively rather than formally coded due to the limited number of responses and their supplementary purpose to expand on closed-ended answers (e.g., selecting ‘Yes’ or ‘No’ and providing reasoning) [66].

## Results

### Formative evaluation

Five adult cannabis educators and five youth participated in the High Alert intervention pre-test, where they offered feedback on the concepts and content of each module and the overall program [30]. In summary, all modules received higher ratings (out of 4) for being informative (3.88), easy to understand (3.85), believable (3.69), and interesting (3.62), compared to personally relevant (3.58) and convincing (3.47) [30]. Module 2 (risks of DUIC) had the highest overall score (3.77) across these categories, followed by Module 1 (effects of cannabis on driving; 3.70), Module 3 (strategies to avoid DUIC; 3.67), and then Module 4 (risky cannabis use; 3.59). Participants’ feedback led to several enhancements in High Alert prior to its implementation: graphics were incorporated for most pages, completion badges were introduced for each module, references were updated, terminology was modified, and technological issues were resolved. The readability scores for the final program corresponded to a 7th-grade reading level (i.e., Flesch Reading Ease Score of 63.7 and a Kincaid Grade Level of 7.2) [30].

### Process evaluation

#### Context.

The pilot randomized controlled trial ran from November 2023 to October 2024 [31]. Two contextual factors identified a priori that may influence DUIC behaviours were assessed and included (i) seeking information on cannabis and/or DUIC and (ii) experiencing any other external factors (i.e., personal, social or environmental). At the 3-month follow-up, 13.5% (n = 7) reported seeking information and provided various comments: “Google impacts,” “I looked into the laws online…”, “I’ve googled the negative side effects…” and “Online training and research...” Likewise, 19.2% (n = 10) reported various external factors influencing their DUIC behaviour, including “home situation,” “new job,” “higher risk insurance rate, so I don’t want to risk an accident,” “due to examination period and new gym schedule,” and “living in the remote north with no taxis.” No statistical differences existed in the proportions of participants across groups reporting information seeking (χ2 (2) = 3.607, p = .165) or external factors (χ2 (2) = 2.588, p = .274). Unfortunately, not all responses indicated whether participants engaged in more or less DUIC as a result of either factor. Another potential contextual factor identified post-hoc [31] was seasonal effects, as cannabis use and impaired driving infractions peak later in the year and decline early into the new year [67,68]. Since most participants were enrolled in late 2023 and completed follow-ups in early 2024, and three mentioned school or icy weather conditions as contextual factors, we suspect timing may have had an effect. However, the impact of these contextual factors remains speculative and warrants further investigation in future studies.

#### Reach.

There were 102 young drivers (55.4% female, mean age = 20.57 years, SD = 1.93) in the pilot trial, with half (n = 52) completing the 3-month follow-up. After the baseline assessment, 37 were computer-randomized to High Alert, 34 to the active control, and 31 to the passive control. A participant flow diagram is presented in [31]*.* No significant baseline differences were found between groups. Notably, 13 participants in High Alert and 10 in the active control did not start their program despite being automatically directed to it after completing the baseline assessment. We suspect some participants closed their web browser before submitting the baseline survey (i.e., did not click submit), resulting in Qualtrics initially deleting their response or not sending the program links. A brief analysis in [31] comparing 3-month follow-up completers with non-completers shows that dropouts had higher baseline rates of cannabis use and DUIC. A deeper investigation for this paper comparing baseline rates in the High Alert group shows that DUIC instances were highest among the 13 participants who did not start the program (mean = 25.4, median = 8) and the eight who began but did not finish it (mean = 27, median = 6.5), compared to the 16 who completed it (mean = 12.4, median = 5). Furthermore, 77% of non-starters (10/13) and 62.5% of non-completers (5/8) indicated using cannabis at least four times per week at baseline, whereas only 31.3% (5/16) of completers reported the same.

#### Dose delivered/Dose received.

Among the 24 participants who started the High Alert program, one did not finish Session 1 (Modules 1 and 2), five did not start Session 2 (Modules 3 and 4), and two started but did not finish Session 2. Two reminder emails containing program links were sent, confirming that access issues were not the reason for non-completion. Session 1 took just under 10 minutes to complete (mean 9.4, median 8.2), and Session 2 took around 12 minutes (mean 12.2, median 9.5). The minimum program completion time was 9 minutes, and the maximum was 51 minutes. As such, 66.7% (n = 16) of participants received the full intervention.

#### Fidelity.

While all modules were completed in the order specified in the trial protocol, the 1-week timing between sessions varied, demonstrating poor fidelity in delivery. Of the 16 participants who completed both High Alert sessions, just 31% (n = 5) adhered to the intended 7-day spacing between them. Two participants completed the sessions on the same day, three completed them 13–18 days apart, five completed them between 20 and 31 days apart, and one completed them 75 days apart. This variability in session timing may have influenced the overall program effect, highlighting potential challenges in adhering to the prescribed schedule.

Engagement demonstrated good fidelity, with participants in Module 1 (n = 24) responding to 100% of the closed-ended questions (e.g., accept/ challenge) and 92% of the free-text questions. Similar engagement rates were observed in Module 2 (91% closed, 89% free-text; n = 23) and Module 3 (100% closed, 93% free-text; n = 18). Module 4 had slightly lower participants (n = 16) and engagement (85% closed, 89% free-text). Free-text responses for reflections and activities were also engaged with as intended. For instance, when participants were requested to provide “a sentence or two” (Module 1), the average response length was 17.6 words, compared to an average response length of 9.1 words when instructed to “jot down your thoughts” (Module 4). One participant reported issues with embedded video links, and another was suspected of speeding through modules. Aside from these, no other issues were identified, demonstrating good fidelity in engagement.

#### Recruitment.

Despite various fraud prevention strategies [69–74], initial public social media posts received numerous fake bot responses, leading us to cease open public recruitment. This hindered our ability to monitor various metrics (e.g., social media shares/likes) and accurately track the number of eligible humans screened. Attempts to use paid advertisements on Facebook and Instagram proved costly and inefficient due to restrictions in the audience targeting options. We could only filter by location (Ontario) and ages (18–24), with no specific interest-based targeting options (e.g., cannabis-related groups) due to Meta’s advertising restrictions. This limited our reach to young cannabis users, resulting in low engagement and very high click costs. Some sites (e.g., Reddit and Snapchat) rejected paid advertisements since the study pertained to cannabis. Private social media posts (e.g., Reddit, Discord) yielded some success and attracted little bot attention. However, skepticism was prevalent across these private forums, with many expressing that the study aimed to identify and punish youth admitting to illegal behaviour. We encouraged eligible participants to privately share the recruitment poster with peers (i.e., passive snowball sampling). However, we could not assess this strategy’s effects due to ethical limits on tracking participant actions. Finally, we used Western University’s mass email recruitment system to invite registered students to participate in November 2023, January 2024, and February 2024. Although this approach proved to be the most effective, it also limited the sample’s diversity by focusing solely on students from a single university. Several other Ontario universities and colleges were invited to support recruitment but declined. We estimate that 83.3% (n = 85) of participants were recruited via Western’s mass email system (38 provided a verified Western email address), while the remaining 16.7% (n = 17) came from social media. In the last two months of recruitment, we set values in the survey URL to identify which social media site/recruitment source respondents came from. This strategy proved useful in identifying the recruitment sources that brought in the most participants while attracting minimal bot attention and is recommended for future implementations.

### Outcome evaluation

#### Behavioural outcomes.

Findings from the primary and secondary behavioural outcomes are reported elsewhere [31]. In summary, High Alert showed the greatest mean reductions in DUIC behaviour over the past 3 months compared to the active and passive controls. These reductions were statistically significant when comparing High Alert with the passive control for total DUIC instances (cannabis and co-use), but not the active control or when examining DUIC from cannabis alone. There were no significant differences in risky cannabis use between groups at the 3-month follow-up [31].

#### Behavioural intentions.

Following the completion of High Alert (n = 16) and the active control (n = 22), significant differences were noted in participants’ expectations of reducing DUIC. In the High Alert group, 94% (15/16) expected a reduction in DUIC, compared to 50% (11/22) in the active control (U = 248.00, z = 2.617, p = .033). Change scores for future DUIC intent showed reductions across all three groups over time. The High Alert group (n = 16) had the largest decrease (mean: −1.31, median: −1.5), followed by the passive control (mean: −0.45, median: −1, n = 16) and then active control (mean: −0.38, median: −0.5, n = 20), although these differences were not statistically significant (H(2)= 4.387, p = .112).

#### Behavioural determinants.

Table 3 outlines the differences in change scores across groups and the proportion of participants showing more favourable scores. Participants’ knowledge scores rose from baseline to post-intervention and were significantly higher among High Alert than the active control (U = 106.50, z = −2.077, p = .039). High Alert participants (n = 16) knowledge increased by 4.56 points (SD = 3.44) on the 27-point scale, while the active control group (n = 22) had a smaller increase of 2.18 points (SD = 2.82). Notably, High Alert participants demonstrated the greatest improvements across other determinants, except for normative beliefs, where the passive control group showed the largest change (−0.40). High Alert participants also had the highest proportion of individuals with improved scores (compared to no change or worsening attitudes) across all variables, except for normative beliefs and self-efficacy to avoid DUIC. However, none of these changes were statistically significant between groups.

**Table 3:** Changes in DUIC determinants.

Determinants	High Alert (n = 16)	Active control (n = 16)	Passive control (n = 20)	Test Statistic
Knowledge score				
Mean change (+)	4.56	2.18	n/a	U = 106.50, z = −2.077, *p* **= .039**
Proportion showing improvements:	100% (n = 16)	77% (n = 17/22)		
Moral wrongness of DUIC				
Mean change (+)	0.31	−0.38	0.05	H(2)= 3.395, *p* = .183
Proportion showing improvements:	37.5% (n = 6)	25% (n = 4)	20% (n = 4)	
Acceptability of DUIC				
Mean change (-)	−0.25	0.13	0.15	H(2)= 4.458, *p* = .108
Proportion showing improvements:	37.5% (n = 6)	12.5% (n = 2)	5% (n = 1)	
Dangerousness of DUIC				
Mean change (+)	0.31	−0.06	0.25	H(2)= 2.290, *p* = .318
Proportion showing improvements:	37.5% (n = 6)	18.8% (n = 3)	30% (n = 6)	
Legal risk of DUIC				
Mean change (+)	0.31	−0.19	0.00	H(2)= 4.960, *p* = .084
Proportion showing improvements:	37.5% (n = 6)	12.5% (n = 2)	20% (n = 4)	
Normative Beliefs				
Mean change (-)	−0.25	−0.19	−0.40	H(2)= 1.051, *p* = .591
Proportion showing improvements:	25% (n = 4)	18.8% (n = 3)	30% (n = 6)	
Self-efficacy to avoid DUIC				
Mean change (+)	0.38	−0.06	0.15	H(2) = 1.425, *p* = .490
Proportion showing improvements:	31% (n = 5)	25% (n = 4)	35% (n = 7)	
Resisting DUIC peer pressure				
Mean change (+)	0.69	0.06	0.00	H(2) = 2.764, *p* = .251
Proportion showing improvements:	43.8% (n = 7)	18.8% (n = 3)	15% (n = 3)	

Variables marked with a (-) indicate that a negative change reflects improvement in attitudes, while variables marked with a (+) indicate that a positive change reflects improvement. DUIC = driving under the influence of cannabis.

### Acceptability

There were 16 participants in High Alert and 22 in the active control that completed the post-test immediately after completing their randomly assigned program. Table 4 displays differences in program content and delivery scores, program recommendations, and perceived impact on DUIC behaviour (results presented above). The High Alert program received a significantly higher rating across all content-related categories except for believability (average 3.63) than the active control (average 2.93), with ratings similar to youth from the pre-test (average 3.66). There wasn’t much difference between the two programs’ averaged delivery scores (3.41 vs 3.29). Notably, 81% (13/16) of people in the High Alert group would recommend this program to their peers, which is significantly higher than the 40% (9/22) who would recommend the active control (U = 94.00, z = −2.568, p = .015). Feedback about High Alert was mostly positive, with 50% (n = 8) of participants providing optional feedback on specific aspects of the program they liked, and 12.5% (n = 2) providing comments on parts they disliked. Positive written examples included “Other participant’s answers,” “knowledge quizzes,” "the phrases to use against peer pressure,” and “the prompts and reflections throughout the module.” The two negative comments were: “Most of the embedded video links didn’t work” and “I felt like the ‘see what other youth answered’ were not real answers, more like model answers.”

**Table 4:** High alert acceptability ratings.

	High Alert Pre-Test (n = 5)	High Alert (n = 16)	Active Control (n = 22)	Test Statistic ^a^
**In your opinion, was the program content:**				
Informative	3.80	3.81	3.05	U = 73.50, z = −3.340, *p* = .002*
Easy to understand	3.84	3.75	3.32	U = 108.50, z = −2.280, *p* = .045*
Convincing	3.48	3.63	2.64	U = 63.00, z = −3.562, *p* = .001*
Interesting	3.60	3.56	2.64	U = 85.50, z = −2.830, *p* = .006*
Believable	3.68	3.50	3.14	U = 136.00, z = −1.286, *p* = .246
Personally Relevant	3.58	3.50	2.77	U = 80.00, z = −3.093, *p* = .004*
Average scores:	3.66	3.63	2.93	–
**In your opinion, was the software/ application used to deliver the program:**				
Easy to use	–	3.38	3.59	U = 200.00, z = −0.834, *p* = .492
Engaging	–	3.44	3.00	U = 118.00, z = −1.860, *p* = .089
Average scores:	–	3.41	3.29	–
**Overall, how likely are you to recommend this program to your peers**				
Very Unlikely	–	–	n = 1	U = 94.00, z = −2.568, *p* = .015*
Unlikely	–	–	n = 6	
Neutral	–	n = 3	n = 6	
Likely	–	n = 10	n = 7	
Very Likely	–	n = 3	n = 2	
**After this program, how often do you think you will drive under the influence of cannabis?**				
More than before	–	n = 1	n = 1	U = 248.00, z = 2.617, *p *= .033*
No change	–	–	n = 10	
Less than before	–	n = 15	n = 11	

Ratings were on a 4-point scale: 1- not at all, 2 – slightly, 3 – moderately, 4 – very.

^a^Analysis excludes the High Alert pre-test responses.

## Discussion

This study presents the evaluation for High Alert using Step 6 of the Intervention Mapping framework and reports findings from the formative, process, outcome, and acceptability evaluations. Given that Step 6 is often underreported compared to earlier steps in this framework, this study extends the current DUIC prevention literature and serves as a practical guide for researchers employing Intervention Mapping for evaluation.

The formative and acceptability evaluations underscore High Alert’s positive reception among youth, with most participants willing to engage with it and recommend it to peers. The program achieved high content ratings that were significantly greater than the various static infographics displayed to the active control. Examining the findings from each evaluation together provides additional insights. For instance, Module 4 on risky cannabis use received the lowest pre-test ratings and participant engagement scores, which suggests a need for potential refinements before future testing and implementations.

The outcome evaluation provided initial support for High Alert’s potential to reduce DUIC behaviours compared to no contact, particularly when considering driving after cannabis co-use. However, no differences were found when examining the DUIC (cannabis-only) variable, prompting a need for additional testing and better outcome variable measurement. High Alert participants demonstrated significantly greater knowledge gains when compared to the active control group and were more likely to report expectations of reducing DUIC afterwards. This suggests that High Alert’s contents and interactive elements (e.g., reflective exercises, real-life cases, multimedia) might have been more effective at reinforcing learning compared to static materials in the active control. However, knowledge gains did not translate into significant differences across other behavioural outcomes, highlighting a common gap between knowledge acquisition and engaging in risky behaviour [50,75,76].

Aside from knowledge, no significant changes were observed in other behavioural DUIC determinants. One explanation is that the theory-based methods and practical strategies used to deliver content (as described in [30]) may not have effectively influenced the targeted determinants. However, High Alert participants demonstrated the greatest improvements in mean change scores and had the highest proportion of individuals with improved attitudes across most determinants, even though they were not statistically significant. Alternatively, the statistical methods used to assess changes in determinants across groups (i.e., analyzing change scores with the Kruskal-Wallis H test or Mann-Whitney U tests) might have been insufficient to detect subtle changes over time. Although these non-parametric tests were chosen a priori due to the large number of Likert-type variables, violations of normality, and their use in similar studies [77–80], future research could benefit from more robust analytical techniques, such as repeated measures mixed modelling, which Teeters and colleagues [11] used to identify changes in perceptions of DUIC dangers over time.

The process evaluation recognized several recruitment and implementation challenges, offering insights for future improvement. Recruitment was hindered by skepticism about the study’s intentions, limited targeting options in paid advertisements, and the prevalence of bot responses on public social media. Bots are increasingly problematic in online research, and many published fraud prevention methods were ineffective in this trial [69–74]. While mass email recruitment was effective and attracted the least bot attention, it limited the sample diversity. Future research should focus on private recruitment strategies and establish early partnerships (e.g., academic institutions, high schools, driving schools) for mass outreach to verified participants. Public social media could be valuable for future widespread implementation but should be avoided in the interim efficacy testing to mitigate bot exposure. Retention was another challenge, with pilot trial attrition (57%) exceeding that of comparable studies [26,81,82] and highest among those reporting frequent cannabis use and DUIC [31]. This echoes findings from previous studies [83] and highlights the need for tailored strategies to improve retention among higher-risk participants. Another issue was adherence to the 7-day spacing between Sessions 1 and 2, a decision informed by expert and youth recommendations [12,13,30]. This split may have led to greater attrition, as 20% (n = 5) of those who started High Alert did not start Session 2. Unfortunately, we were unable to identify why participants did not adhere to or start the second session. No technical issues were reported, and emails may have been flagged as junk or ignored until the reminder was sent. Future research could also send session reminders via text messaging [84] and explore whether redesigning High Alert into a single session leads to better adherence and fewer dropouts than two separate sessions [85].

Integrating findings from all four evaluations offers a holistic view of High Alert’s initial implementation, preliminary efficacy, and areas for improvement in program delivery and testing. Reporting these findings from Step 6 provides valuable guidance for researchers conducting similar studies, enabling them to develop comprehensive evaluations and proactively address many of the implementation challenges we faced. Step 6 evaluations are often underreported [28–30,32–43], yet they are essential for understanding the complexities of program delivery. Without this data, there is a risk of misinterpreting the effect of interventions, as it overlooks critical elements such as fidelity, participant engagement, and contextual influences. By underscoring the importance of these evaluations, we advocate for more detailed and transparent reporting in intervention research, especially for studies targeting high-risk behaviours like youth cannabis use and DUIC.

### Limitations

Despite the strengths of this study, which include using a comprehensive, evidence-based evaluation framework, gathering acceptability findings that can be compared across pre-test and pilot trial participants, and the ability to identify implementation challenges and refine the program before a larger randomized controlled trial, several limitations must also be considered. First, technical issues arose with the Qualtrics surveys and randomization, which may have resulted in a smaller sample size and potential group crossover. Some participants likely closed their browsers before submitting the baseline survey, leading to incomplete responses being deleted and reducing the potential sample size. Although settings were modified soon after to prevent future deletion, some responses continued to be flagged as incomplete and prevented the automatic sending of program links. Efforts were made to identify these participants and manually resend the links, but some cases may have been missed, further reducing the sample. Additionally, during the first week of recruitment, the Qualtrics randomizer failed to record assignments for about 15 participants, requiring manual identification based on program engagement. This increased administrative work and the risk of group crossover (e.g., incorrectly labelling participants as passive control if they didn’t engage). The small sample resulting from these technical issues limits the generalizability of the findings and may have affected the statistical power to detect significant differences in some outcomes. Additionally, the recruitment challenges, including participant skepticism, limited paid advertising options, and bot interference, likely introduced bias and limited the representativeness of the sample.

Second, while Qualtrics surveys were password-protected with participants’ unique codes, the CIAS platform for High Alert lacked this feature. This led to some mismatched codes across platforms, likely from participant typos or bot entries. Since CIAS data were anonymous and only linked via the unique code (with no email addresses stored), we were unable to verify participation and differentiate between legitimate and bot responses for mismatched codes, leading to the exclusion of some data.

Third, participants received immediate access to the first High Alert session after randomization, but the link to the second session was automatically sent one week later, regardless of engagement. While this approach was initially chosen to reduce administrative burden and human errors in sending links, it likely contributed to poor program delivery fidelity. Some participants, having received the link to the second session early, may have completed both sessions in one day, while others experienced large gaps between sessions due to delays in starting the second session.

Fourth, participants’ written responses were reviewed and presented descriptively due to the limited number of responses and their supplementary purpose [66]. However, future trials with larger samples and additional written responses should consider formal qualitative analysis, such as formative or summative content analysis [86], to provide additional insights that may have been missed. Lastly, although the acceptability findings indicate a positive reception of the program, these results may not fully reflect the views of participants who dropped out before completing the program. This is especially relevant for outcome evaluations, as most dropouts reported higher cannabis use and DUIC at baseline.

### Future directions

The comprehensive evaluation of the High Alert intervention provided valuable insights into the program’s preliminary efficacy, pilot implementation, and areas for refinement. Key identified improvements include the need for a more rigorous assessment of contextual factors (e.g., clarifying how these factors influence DUIC engagement), enhancing recruitment to mitigate bots and increase the appeal to participate, addressing technical issues, improving retention strategies for high-risk participants, monitoring fidelity rates more effectively, enhancing DUIC outcome measures, and employing more robust analytical techniques to evaluate changes in behavioural determinants. These insights are essential for optimizing the program before further testing. According to the Intervention Mapping framework, Step 5 can be revisited if the program demonstrates efficacy, facilitating additional planning in program adoption, implementation, and sustainability [27]. This proactive evaluation approach establishes a strong foundation for future studies to evaluate digital smartphone interventions (including High Alert) in larger randomized controlled trials and, if found efficacious, to evaluate their effectiveness in real-world settings. Such information is crucial, especially given the common limitations associated with smartphone interventions, including the rapid pace of technological development, challenges with user engagement, technical issues, and the need to comply with regulatory, ethical, and security requirements [23,24,45,46]. Addressing these limitations proactively can enhance the effectiveness of interventions and ensure that digital programs like High Alert can be successfully implemented and sustained at scale. While this pilot study intentionally recruited youth who recently engaged in DUIC to allow for immediate assessment of DUIC behaviour change, High Alert has the potential for broader applications. Expanding to all youth, regardless of current cannabis or DUIC behaviours, could foster a widespread shift in attitudes and norms around the risks of DUIC and better position peers to be environmental agents of change [87–89]. This is advantageous given youth’s lack of awareness of the risks of DUIC [50,51,59,90,91] and can be explored in future research.

## Conclusions

Comprehensive evaluations are crucial for programs like High Alert to support ongoing improvements, assess implementation, evaluate program objectives, and ensure participant acceptability for broader applications. Findings from this evaluation offer valuable insights for refining the program and support the design of a larger randomized controlled trial. If proven effective, High Alert may serve as a practical tool for reducing DUIC among Canadian youth and help address this public health issue.
